# Buprenorphine enhances *Streptococcus mutans* virulence and biofilm formation

**DOI:** 10.1080/20002297.2026.2652173

**Published:** 2026-04-09

**Authors:** Marlus S. Pedrosa, Luca L. Iannaccone, Charles S. Schuls, Apoena A. Ribeiro

**Affiliations:** a Department of Diagnostic Sciences (Cariology and Microbiology), Adams School of Dentistry, University of North Carolina at Chapel Hill, Chapel Hill, NC, USA; b Department of Orofacial Sciences, School of Dentistry, University of California, San Francisco, CA, USA; c College of Arts and Sciences, University of North Carolina at Chapel Hill, Chapel Hill, NC, USA

**Keywords:** Buprenorphine, *Streptococcus mutans*, biofilms, genetic competence, oral health

## Abstract

**Background:**

Buprenorphine is widely prescribed for opioid use disorder (OUD). In 2022, the U.S. FDA issued a safety warning on dental diseases associated with buprenorphine. While reports implicate increased caries risk, the microbial mechanisms remain unclear.

**Objective:**

To evaluate whether buprenorphine directly modulates *Streptococcus mutans* (*S. mutans*) virulence traits relevant to cariogenesis.

**Design:**

*S. mutans* UA159 were exposed to buprenorphine. Planktonic growth, acidogenicity, acid tolerance, aggregation, and carbohydrate utilization were assessed. Biofilm biomass and extracellular polymeric substance (EPS) production were quantified in hydroxyapatite disc–based monospecies and saliva-derived microcosm models. Biofilm architecture was evaluated using fluorescence *in situ* hybridization (FISH). The expression of competence- and biofilm-associated genes (*comC, comX, gcrR, gtfB,* and *gtfC*) was measured by RT-qPCR.

**Results:**

Buprenorphine did not affect planktonic growth, acid production, or carbohydrate metabolism. However, it increased biofilm biomass and EPS production. FISH imaging revealed denser matrix-rich biofilms with closer spatial integration of *S. mutans*. Gene expression showed upregulation of *comC, comX, gcrR, gtfB,* and *gtfC*, indicating enhanced quorum sensing, stress adaptation, and matrix synthesis.

**Conclusions:**

Buprenorphine promoted a biofilm-specific virulence program in *S. mutans*, fostering thicker, EPS-rich biofilms without altering planktonic physiology. These findings provide a mechanistic rationale for buprenorphine's association with caries risk.

## Key messages

Buprenorphine triggered biofilm-specific virulence responses in *Streptococcus mutans*, increasing biofilm mass and extracellular matrix production without affecting growth or metabolism in planktonic culture. These mechanistic findings support clinical observations of severe caries in patients receiving buprenorphine therapy and underscore the need for proactive oral-health prevention in this population, as highlighted by the 2022 U.S. Food and Drug Administration safety warning.

## Introduction

The global burden of opioid use disorder (OUD) has led to the widespread use of medication-assisted therapies, including buprenorphine, a partial μ-opioid receptor agonist commonly prescribed for long-term management of OUD [1,2]. While buprenorphine has demonstrated significant benefits in reducing opioid dependence and associated mortality [3,4], emerging reports suggest a potential link between opioid use and deterioration of oral health, including an increased prevalence of dental caries, periodontal disease, and tooth loss [5–11]. In January 2022, the FDA issued a warning about dental problems associated with buprenorphine dissolved in the mouth to treat OUD [10,12]. Despite these observations, the underlying biological mechanisms connecting opioid therapy and oral disease remain poorly understood.

Dental caries is a dysbiotic disease driven by the activity of cariogenic bacteria such as *Streptococcus mutans* (*S. mutans*), which colonize tooth surfaces, form biofilms, produce extracellular polysaccharides (EPS) and generate acid as a metabolic byproduct of dietary's carbohydrate fermentation [13–15]. The ability of *S. mutans* to produce acid (acidogenicity) and to thrive in low pH environments (aciduricity) are key virulence traits that contribute to its dominance in cariogenic biofilms and its role in disease progression [16,17]. These processes lead to localized demineralization of enamel and dentin, ultimately resulting in cavity formation [13,14]. The formation and persistence of biofilms are critical for *S. mutans* pathogenicity, as they protect against environmental stressors, antimicrobial agents, and host immune defenses [13–15,18].

Previous research has shown that certain medications can alter the oral environment by reducing salivary flow, shifting the pH or disrupting the microbial balance, factors that collectively promote the development of cariogenic biofilms and oral dysbiosis [19–22]. Opioid addiction, in particular, exacerbates these risks by directly impairing salivary secretion, thereby increasing susceptibility to dental caries and periodontal disease [23,24]. These oral health consequences are not only linked to behavioral factors such as neglect and financial constraints but are also driven by the direct physiological effects of opioid use on oral tissues and the microbiota [25,26]. Despite the high prevalence of xerostomia and other drug-induced oral alterations [23,24], data on dental disease in this population remain limited. However, it is clear that opioid users experience significantly higher rates and severity rates of dental caries and periodontal disease compared to the general population [27].

Although the detrimental effects of buprenorphine on oral health have been reported, the existing literature primarily consists of case reports [6,9], research letters [7] and case‒control studies [8], with limited mechanistic insight [28]. To the best of our knowledge, the specific impact of buprenorphine on *S. mutans* physiology and biofilm formation has not been thoroughly characterized [28], and a single study aimed to fill that gap by investigating the potential mechanisms through which local buprenorphine exposure influences dental biofilm development [29]. These findings suggest that sublingual administration of buprenorphine significantly increases its accumulation in the salivary glands, sustaining elevated levels of buprenorphine in the oral cavity and creating conditions that may enhance *S. mutans* biofilm formation [29]. However, the extent to which increased oral exposure to buprenorphine contributes to dental adverse effects remains unclear.

Understanding how buprenorphine alters the virulence traits of *S. mutans* is critical for uncovering potential microbial mechanisms underlying the increased risk of dental caries in patients undergoing OUD treatment. Although individuals with OUD are already vulnerable to poor oral health outcomes, the specific impact of buprenorphine on cariogenic bacteria remains poorly defined. In this study, we investigated whether buprenorphine influences *S. mutans* growth kinetics, acidogenicity and acid tolerance, as well as its ability to form biofilms and synthesize EPS. In addition, we assessed the expression of genes involved in competence and matrix production to determine whether buprenorphine promotes a transcriptional shift toward a more virulent, biofilm-enhancing phenotype.

## Materials and methods

### Drug reconstitution

Buprenorphine hydrochloride (Supelco, Cat# PHR1729) was dissolved in molecular-grade water (10 mg/mL; ~20 mM) and sterilized by filtration through a 0.2 µm membrane filter. This stock solution was subsequently diluted in the indicated assays to achieve the final working concentrations used in each experiment.

### Bacterial culture

*S. mutans* UA159 (serotype c) were streaked on brain heart infusion (BHI) agar plates, and a single colony was grown overnight in BHI at 37 °C and 5% CO_2_.

### Growth assays

Growth assays were performed as described elsewhere [30]. Briefly, overnight cultures were centrifuged and washed twice with 1× phosphate-buffered saline (PBS) to remove trace compounds from the culture media. The cells were resuspended and normalized by optical density at 600 nm (OD_600_) in test media (OD_600_ = 0.1). The cultures were grown at 37 °C and 5% CO_2_ without shaking. The OD_600_ was recorded using a microplate reader (SpectraMax M2e).

### Acidogenic potential and acid tolerance

The culture media were inoculated with an OD_600_ of 0.1 of the bacterial strain. The acidogenic potential was assessed by monitoring the pH drop in the culture medium using a pH meter (FiveEasy Benchtop F20 pH/mV Meter, Mettler Toledo) [31]. Acid tolerance tests [31] were performed using BHI broth with pre-adjusted pH using lactic acid (13.42 M) to adjust the pH of the medium to pH 5, pH 6 and pH 7. The pre-adjusted pH media was inoculated with an OD_600_ of 0.1 of the bacterial strains, and 200 μL was transferred to a 96-well plate to study bacterial growth under acidic conditions. Culture grew at 37 °C and 5% CO_2_ without shaking. The OD_600_ was recorded every 1 h using a microplate reader (SpectraMax M2e).

### Colony-forming unit (CFU) quantification

At the desired time points (as indicated in the figure legends), the cultures were vortexed to ensure homogeneity and 100 µL of each planktonic bacterial suspension was serially diluted 10-fold in phosphate-buffered saline (PBS). A 20 µL aliquot of each dilution was plated onto BHI agar plates. The plates were incubated at 37 °C in 5% CO_2_ for 24 h before CFU counting. Results were expressed as CFU per milliliter (CFU/mL). As for hydroxyapatite (HA) discs, cultures were then transferred into sterile tubes containing 1 mL PBS and vortexed for 30 s to detach biofilm bacteria. The resulting suspension was serially diluted, and a 7 µL aliquot of each dilution was plated onto BHI agar plates. The enumeration CFU per cm² (CFU/cm²) were calculated by multiplying the number of colonies by the dilution factor and dividing by the product of the plated volume (in mL) and the total specimen surface area (1.1654 cm²), which includes the top, bottom and lateral surfaces of the specimens.

### Bacterial aggregation assay

Bacterial aggregation was assessed using an OD_600_-based turbidity protocol previously described [32]. Cultures (5 mL) were incubated statically in sterile borosilicate glass tubes at 37 °C in 5% CO₂ for 24 h. Following incubation, the tubes were gently inverted to disrupt loosely attached cells and vortexed briefly (10 s) to resuspend the aggregates. The turbidity of the resulting suspensions was measured spectrophotometrically, and higher OD values reflected greater aggregate mass retained after resuspension. All the conditions were tested using three independent biological replicates, each measured with three technical replicates and the values were normalized to the untreated control.

### Mono-species biofilm

Mono-species biofilms of *S. mutans* were established using BHI broth supplemented with 0.2% sucrose (BHI-sucrose) to promote biofilm development. Salivary pellicles were formed by immersing HA in filtered saliva overnight at room temperature. Overnight cultures of *S. mutans* were grown in BHI at 37 °C in 5% CO_2_. Cultures were normalized to an OD_600_ of 0.1 in fresh BHI-sucrose, and 1 mL of inoculum was added to each well of a sterile 48-well flat-bottom polystyrene plate on saliva-coated HA discs mounted in a vertical position. The plates were incubated at 37 °C in 5% CO_2_ for 24, 48 or 72 h without agitation.

### Microcosm biofilm using human saliva

Stimulated whole saliva was collected from ten healthy adult donors (average age: 23 years; range: 20–25 years) who met the following inclusion criteria: normal salivary flow (stimulated > 1 mL/min; unstimulated > 0.3 mL/min), a history of caries without active lesions (i.e. no white spot or cavitated lesions), no signs of gingivitis or periodontitis (e.g. gum bleeding or tooth mobility) and no antibiotic use in the three months prior to collection. Donors were instructed to avoid toothbrushing for 24 h and refrain from food or drink intake for at least 2 h before saliva donation [33]. The collected saliva was pooled, mixed with glycerol to a final concentration of 70% and divided into two portions. One portion was stored at −80 °C for later use as a biofilm inoculum, while the other portion was used to prepare sterile salivary pellicles by centrifugation (12,000 × *g*, 15 min), followed by filtration through a 0.22 μm membrane (Thermo Scientific™ Nalgene™ Rapid-Flow™) to remove bacteria. The resulting bacteria-free saliva was stored at −80 °C until use [34]. Salivary pellicles were formed by immersing hydroxyapatite (HA) discs in filtered saliva overnight at room temperature. Microcosm biofilms were cultured using McBain medium prepared with 0.2% sucrose (Fisher Scientific, Cat# S2500) as previously described [34–36], adjusted to pH 7.0 and sterilized. Sucrose-supplemented McBain medium was used during both biofilm initiation and all subsequent incubation steps to support EPS-dependent biofilm formation. For biofilm inoculation, HA were placed vertically in 48-well plates and 1 mL of McBain medium containing saliva-glycerol stock diluted 1:50 was added per well. The plates were incubated at 37 °C in 5% CO_2_ for 24, 48 or 72 h. At each 24-h interval, the disks were transferred to new plates containing fresh sucrose-supplemented McBain medium (without saliva). Biofilms were collected at the indicated time points to allow for robust microcosm biofilm formation [34,35].

### Biofilm biomass quantification by crystal violet assay

Biofilms were gently washed with PBS to remove planktonic cells and stained with 0.1% crystal violet for 15 min. Excess stains were removed by washing, and bound dye was solubilized in 30% acetic acid. The biofilm biomass was quantified by measuring the absorbance at 570 nm (OD_570_) using a microplate reader (SpectraMax M2e). All experiments were performed in triplicate with at least three independent biological replicates.

### EPS quantification

EPS extraction was performed as previously described with slight modifications [37,38]. Mature biofilms were developed on HA discs using saliva-derived inoculum as described above. After incubation, non-adherent cells were gently removed by washing the HA discs twice with 1× DPBS. To extract the extracellular polysaccharides, the biofilm biomass was collected, suspended in 0.1 M sodium hydroxide (NaOH) and vortexed briefly. The suspension was incubated overnight at 4 °C and then centrifuged at 10,000 × *g* for 10 min at 4 °C to collect the EPS-containing supernatant. Then, EPS was quantified using the phenol-sulfuric acid method in a microplate format [38]. Briefly, 50 μL of the EPS-containing supernatant was mixed with 150 μL of concentrated sulfuric acid (Sigma, Cat. 258105) and 30 μL of 5% phenol (Sigma, Cat. P1037). The mixture was incubated for 30 min at room temperature, and the absorbance was measured at 490 nm using a microplate reader (SpectraMax M2e). A glucose (D-glucose, Sigma Cat. G8270) standard curve was used for quantification.

### Fluorescence *in situ* hybridization (FISH) and EPS staining of oral biofilms

*In vitro* oral biofilms were developed on HA discs in the previously described microcosm model. At 48 h, biofilms were fixed and processed for multiplex fluorescence staining to visualize *S. mutans*, total bacterial populations and EPS. Briefly, HA discs were gently rinsed three times by immersion in sterile 0.9% NaCl for 1 min each and then transferred to sterile 48-well flat-bottom plates. The biofilms were fixed with 0.5 mL of 4% paraformaldehyde in PBS for 1 h at RT, followed by a rinse with sterile saline. For permeabilization, the discs were incubated for 15 min at 37 °C in lysozyme (≥70,000 U/mL, Sigma-Aldrich) prepared in 100 mM Tris–HCl buffer (pH 7.5) and then rinsed again in saline. FISH hybridization was performed using specific oligonucleotide probes targeting either the 16S rRNA gene of all eubacteria (EUB338; 5′-ACTCCTACGGGAGGCAGCAG-3′, labeled with FAM) or *S. mutans* (MUT590; 5′-ACTCCAGACTTTCCTGAC-3′, labeled with Cy3), both ordered from Integrated DNA Technologies (IDT). The discs were pre-incubated for 15 min at 46 °C in hybridization buffer consisting of 20 mM Tris–HCl (pH 7.5), 0.9 M NaCl, 0.01% SDS, and 20% formamide. Hybridization was carried out in the same buffer containing 5 µg/mL of each probe, with 200 µL added per well and incubated at 46 °C for 180 min in the dark. The discs were then washed in a washing buffer composed of 20 mM Tris–HCl (pH 7.5), 280 mM NaCl, 5 mM EDTA, and 0.01% SDS at 48 °C for 15 min, followed by a rinse with physiological saline. EPS were stained using Concanavalin A conjugated to Alexa Fluor™ 405 (Thermo Fisher Scientific, Cat. C56126). The discs were incubated in a staining solution (50–100 µg/mL in PBS, as per the manufacturer's instructions) for 30 min at room temperature in the dark and rinsed gently with PBS to remove unbound dye. Each disc was then transferred, biofilm-side down, onto a glass-bottom dish (VWR, Cat. 734-2904) containing 20 µL of PBS to maintain hydration. Images were acquired using an Andor Dragonfly spinning disk confocal microscope (Oxford Instruments, UK) equipped with a Leica HC PL APO 20×/0.75 objective and an sCMOS Andor Zyla camera (Oxford Instruments, UK). The following laser and emission settings were used: 405 nm excitation with ~450–460 nm emission for EPS (Alexa Fluor 405), 488 nm excitation with ~520 nm emission for total bacteria (FAM), and 550 nm excitation with ~570 nm emission for *S. mutans* (Cy3). Sequential scanning was applied to prevent spectral overlap between fluorophores. Z-stacks were collected and processed as maximum-intensity projections for display. Images were acquired as 3 × 3 stitched tiles using 40 µm pinholes.

### RNA extraction and quantitative real-time PCR (RT-qPCR)

To quantify buprenorphine-responsive transcription under controlled growth conditions, RT-qPCR was performed using planktonic *S. mutans* cultures. Planktonic cultures were used to ensure uniform drug exposure and consistent RNA yield across biological replicates. *S. mutans* cultures were grown in BHI in the presence or absence of 100 μM buprenorphine. Overnight cultures were diluted to an OD_600_ of 0.1 and incubated at 37 °C under microaerophilic conditions. Accordingly, all RT-qPCR data are labeled as ‘planktonic’ throughout the Results and figure legends. At 4, 6, 8, and 24 h post-treatment, 5 mL of culture was collected and centrifuged at 12,000 × *g* for 2 min. The bacterial pellets were resuspended in 100 μL of freshly prepared lysozyme solution (10 mg/mL in TE buffer) and incubated at room temperature for 10 min. Then, 1 μL of 10% SDS was added, and the samples were gently mixed and incubated for an additional 5 min to ensure cell lysis. Total RNA was extracted using the PureLink™ RNA Mini Kit (Invitrogen, Cat.# 12183025) following the manufacturer's protocol, including on-column DNase I treatment (PureLink DNase Set, Invitrogen, Cat.# 12185010) to remove genomic DNA contamination. The RNA concentration and purity were determined using a NanoDrop One spectrophotometer. For cDNA synthesis, 1 μg of total RNA was reverse-transcribed using the High-Capacity cDNA reverse transcription kit with an RNase inhibitor (Applied Biosystems, Cat.# 43-688-14), according to the manufacturer's instructions. Reactions were performed in a total volume of 20 μL using random primers, and the cDNA was stored at –20 °C until use. Quantitative PCR was performed using Power SYBR™ Green PCR Master Mix (Applied Biosystems, Cat.# 4309155) in 384-well optical plates (Applied Biosystems, Cat# AB1384) and the reactions were run on a QuantStudio 6 Flex Real-Time PCR System (Applied Biosystems). Gene-specific primers were validated for efficiency and specificity; all reactions were run in technical duplicates using 10 ng of cDNA per reaction. Relative gene expression was calculated using the 2^–ΔΔCt^ method, with normalization to 16S rRNA as the reference gene. The primer sequences are listed in Table 1. Each experiment was conducted using three independent biological replicates. Statistical analysis was performed on Δ*C_t_* values using an unpaired two-tailed Student's *t*-test.

**Table 1.** Function and primer sequences of target genes used in RT-qPCR analysis.

Target gene	Function	F/R	Sequence 5′-3′	Reference
*16S*rRNA	Housekeeping gene	F	CACACCGCCCGTCACACC	[39]
		R	CAGCCGCACCTTCCGATACG	
*comC*	The comC gene in *S. mutans* encodes the precursor of the competence-stimulating peptide (CSP), a key component in quorum sensing and biofilm formation		GACTTTAAAGAAATTAAGACTG	[40]
			AAGCTTGTGTAAAACTTCTGT	
*comX*	Alternative sigma factor regulating late competence genes; key regulator in quorum sensing and genetic transformation	F	CGTCAGCAAGAAAGTCAGAAAC	[39]
		R	ATACCGCCACTTGACAAACAG	
*dexA*	Encodes a debranching glucan hydrolase; breaks down branched glucans, contributing to biofilm remodeling and dispersal	F	AGGGCTGACTGCTTCTGGAGT	[41]
		R	AGTGCCAAGACTGACGCTTTG	
*gcrR*	Response regulator that represses *gtfC*; contributes to acid sensitivity, biofilm modulation and competence regulation in S. mutans	F	ACCAGAGATGGACGGGTATG	[41]
		R	CACGATAGGTAGTGTCATTTTTAG	
*gtfB*	Glucosyltransferase B; synthesizes water-insoluble glucans from sucrose, important for biofilm matrix formation and cariogenicity	F	AGCCATGCGCAATCACAGGTT	[42]
		R	CGCAACGCGAACATCTTGATCAG	
*gtfD*	Encodes a glucosyltransferase (GTF-S) enzyme that synthesizes water-soluble glucans. These glucans play a role in the formation of biofilms and the adhesion of *S. mutans* to tooth surfaces	F	TTGACGGTGTTCGTGTTGAT	[43]
		R	AAAGCGATAGGCGCAGTTTA	

F, forward sequence; R, reverse sequence.

### Statistical analysis

The data were analyzed using GraphPad Prism (version X, GraphPad Software, USA). The normality of the data was assessed prior to analysis. The colony-forming unit (CFU) counts were normalized to the surface area and expressed as CFU/cm^2^. To reduce variability and facilitate comparisons across experimental conditions, CFU/mL or CFU/cm^2^ values were log_10_-transformed before statistical analysis. Differences of approximately one log_10_ unit or greater were considered biologically meaningful and are highlighted in the figures. Statistical comparisons among groups were performed using two-way ANOVA followed by Tukey's multiple comparisons test or Student's *t*-test, as appropriate. All experiments were performed in triplicate with at least three independent biological replicates. The data are presented as mean ± standard deviation (SD) and statistical significance was defined as *p* < 0.05. Asterisks indicate levels of significance as follows: *p < 0.05, **p < 0.01, ***p < 0.001 and ****p < 0.0001.

## Results

### Buprenorphine at physiological levels does not affect *S. mutans* growth and viability

**Figure 1.**
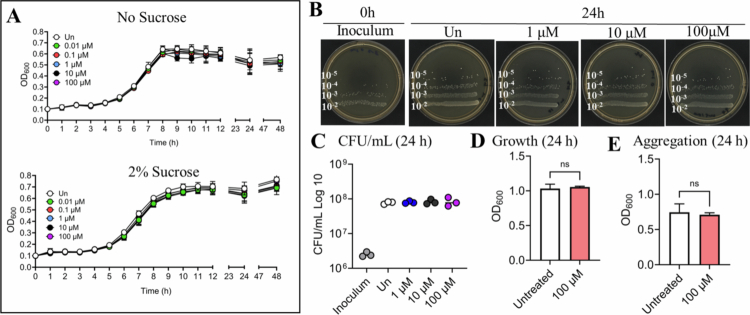
Buprenorphine does not alter *S. mutans* growth, viability or aggregation. (A) Growth curves of *S. mutans* cultured in BHI medium without (top) or with 2% sucrose (bottom), in the presence of increasing concentrations of buprenorphine (0.01–100 μM), monitored over 48 h by OD600. (B) Representative culture plates used for CFU/mL determination. (C) Quantification of colony-forming units (CFU/mL) at 24 h. (D) Total bacterial growth (OD600) after 24 h incubation. (E) Bacterial aggregation assay after 24 h under static conditions. Data represent mean ± SD from three biological replicates performed in duplicate. Statistical comparisons were performed using one-way ANOVA with Dunnett's multiple comparisons test (A–C) or an unpaired two-tailed Student's *t*-test (D–E). *ns* = not significant.

First, we tested the ability of *S. mutans* UA159 to grow under different concentrations of buprenorphine (Sigma, Cat# PHR1729) in BHI supplemented or not with 2% sucrose (Fisher Scientific, Cat# S2500) for up to 48 h. The bacteria exhibited similar growth patterns in the presence and absence of buprenorphine (Figure 1A). To further confirm this observation, we plated bacterial cultures treated with the highest concentrations of buprenorphine (1 μM, 10 μM and 100 μM) and assessed their viability by colony-forming unit (CFU) enumeration. Consistent with the OD_600_ (Figure 1A and D), the CFU counts at 24 h revealed no significant differences between the treated and untreated samples (Figure 1B and C), further supporting the conclusion that buprenorphine does not affect bacterial viability under these conditions. Similarly, aggregation assays showed comparable OD_600_ values between the treatment groups (Figure 1E), suggesting that buprenorphine does not alter bacterial clumping or sedimentation behavior at 24 h.

### Buprenorphine does not affect *S. mutans* acidogenicity or acid tolerance

**Figure 2.**
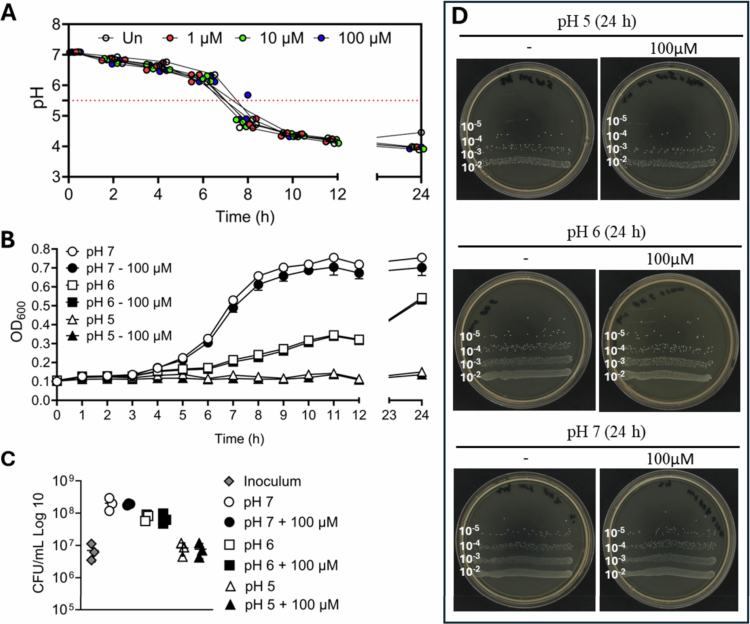
Buprenorphine does not alter acid production or acid tolerance in *Streptococcus mutans*. (A) Acid production by *S. mutans* was assessed by measuring the pH of the culture medium over time in the presence or absence of buprenorphine (1, 10 or 100 µM). The red dotted line at pH 5.5 indicates the critical threshold for enamel demineralization. Data represent the mean ± SD from three independent biological replicates. (B) Growth curves of *S. mutans* in BHI adjusted to pH 7, pH 6 and pH 5, with or without 100 µM buprenorphine. (C) CFU/mL of *S. mutans* after 24 h incubation at pH 5, 6 or 7 in the presence or absence of 100 µM buprenorphine. (D) Representative culture plates used for CFU/mL determination. Data are presented as mean ± SD from three independent biological experiments.

We analyzed pH changes in bacterial cultures over time to assess whether buprenorphine affects the acid-production capacity of *S. mutans.* We observed that buprenorphine treatment could not significantly alter the kinetics of acidogenicity by the bacterial strains (Figure 2a). To determine whether buprenorphine influences the acid tolerance of *S. mutans*, we monitored the optical density OD_600_ over 24 h in *S. mutans* grown in BHI at pH 7, pH 6 and pH 5, with or without 100 µM buprenorphine. The bacteria exhibited robust growth at pH 7, moderate growth at pH 6 and markedly impaired growth at pH 5 (Figure 2C). Importantly, across all strains and pH conditions, the presence of buprenorphine did not significantly affect growth kinetics. These findings indicate that buprenorphine does not directly influence *S. mutans* acid tolerance. To further validate it, we quantified CFU/mL in *S. mutans* at 24 h of incubation in BHI at pH 7, pH 6 and pH 5, with or without 100 µM buprenorphine (Figure 2C and D). *S. mutans* exhibited high viability at pH 7, with CFU counts close to or above 10^8^​​​​​ CFU/mL, regardless of buprenorphine treatment. At pH 6, viability was moderately reduced, while at pH 5, a further drop in CFU counts was observed across all strains. However, under no conditions did buprenorphine significantly alter cell viability relative to that of the untreated controls. These data confirm that buprenorphine does not impair or enhance bacterial survival, even under acidic stress.

### Buprenorphine has minimal impact on carbohydrate metabolism in *S. mutans*

**Figure 3.**
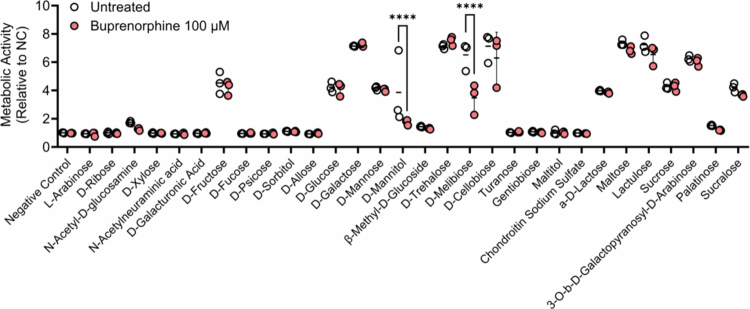
Buprenorphine has minimal impact on carbohydrate metabolism in *S. mutans* Wild-type Strain. Wild-type (WT) *S. mutans* was cultured in the presence or absence of 100 μM buprenorphine and assessed for metabolic activity using the PreBioM1™ substrate utilization assay. The metabolic activity for each carbohydrate substrate was normalized to that of the negative control (NC). Each dot represents a technical replicate; the data are from three independent biological replicates. Statistical analysis was performed using two-way ANOVA with Šídák's multiple comparisons test. ****p < 0.0001.

To assess whether buprenorphine influences carbohydrate metabolism in *S. mutans*, we performed a substrate utilization assay using the PreBioM1™ panel in the presence or absence of 100 μM buprenorphine (Figure 3). Metabolic activity was quantified across a range of carbohydrate substrates and normalized to that of the negative control. Overall, we observed no major differences in substrate utilization between untreated and buprenorphine-treated samples, indicating that buprenorphine does not broadly impair carbohydrate metabolism in *S. mutans* under these conditions. However, two substrates (D-melibiose and D-cellobiose) showed significantly increased metabolic activity in the buprenorphine-treated cultures compared to untreated controls (*p* < 0.0001). These findings suggest that while buprenorphine does not disrupt central carbohydrate metabolic pathways, it may selectively enhance utilization of specific disaccharides, which could contribute to altered biofilm dynamics in the presence of this drug.

### Buprenorphine enhances biofilm biomass and EPS production in *S. mutans* monospecies biofilms

**Figure 4.**
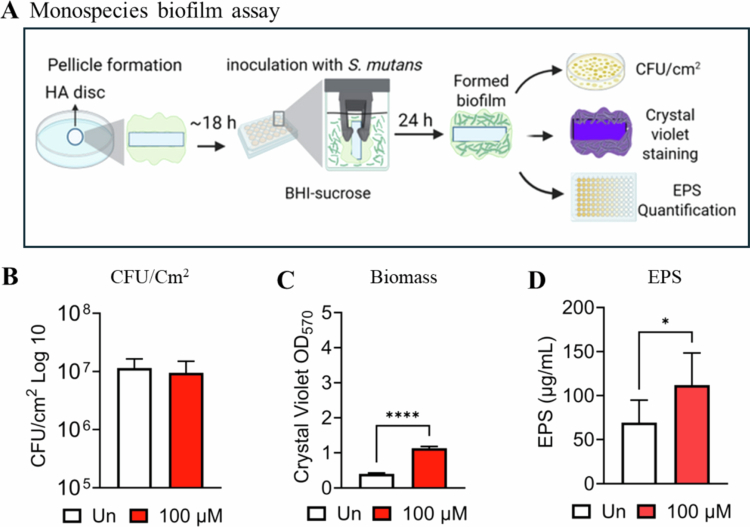
Buprenorphine enhances EPS production and biofilm biomass in *S. mutans* monospecies biofilms. (A) Schematic of the monospecies biofilm assay. HA discs were coated with salivary pellicle and incubated for 18 h, followed by inoculation with *S. mutans* in BHI-sucrose media. After 24 h of biofilm growth under static conditions, biofilms were quantified for: (B) colony-forming units (CFU/cm^2^), (C) total biomass (crystal violet staining) and (D) extracellular polymeric substances (EPS). Data represent means ± SD from three biological replicates performed in duplicate. Statistical comparisons were made using unpaired two-tailed *t*-tests.

To investigate the impact of buprenorphine on *S. mutans* biofilm development, we utilized a monospecies biofilm model in which HA discs were coated with salivary pellicle, inoculated with *S. mutans* in BHI-sucrose medium and incubated under static conditions for 24 h (Figure 4A). The quantification of viable bacteria within the biofilm showed no significant difference in colony-forming units (CFU/cm^2^) between the untreated and buprenorphine-treated conditions (Figure 4B), indicating that buprenorphine does not affect bacterial viability or surface colonization. However, crystal violet staining revealed a significant increase in total biofilm biomass in cultures treated with 100 μM buprenorphine (Figure 4C; *p* < 0.0001). Furthermore, measurement of EPS showed that buprenorphine significantly increased EPS production compared to untreated controls (Figure 4D; *p* < 0.05). These results demonstrate that buprenorphine promotes biofilm formation by enhancing both biofilm mass and EPS, independent of changes in bacterial viability.

### Buprenorphine promotes biofilm biomass and EPS production in a saliva-derived microcosm model

**Figure 5.**
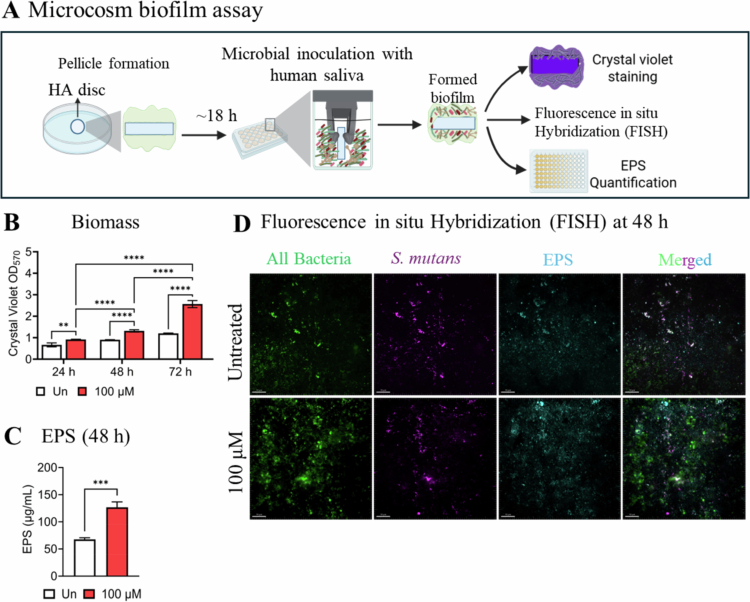
Buprenorphine promotes biofilm formation and EPS production in a microcosm biofilm assay. (A) Schematic representation of the *in vitro* biofilm model used to evaluate microbial colonization on restorative materials. Created with BioRender.com. Composite resin or HA specimens (7 × 1.8 mm) were fabricated and UV sterilized before use. Stimulated saliva was collected from healthy adult donors, pooled and processed to obtain a standardized microbial inoculum. All specimens were incubated overnight (~18 h) in filtered human saliva to promote salivary pellicle formation on their surfaces. Microcosm biofilms were initiated by inoculating pellicle-coated specimens with McBain medium diluted 1:50 with processed unfiltered saliva. The biofilms were incubated at 37 °C under aerobic conditions with 5% CO_2_. (B) Quantification of biofilm biomass in a saliva-derived microcosm model grown on HA discs for 24, 48 and 72 h, assessed by crystal violet staining (OD_570_), in the presence of 100 µM buprenorphine. (C) Quantification of EPS at 48 h. Data represent mean ± SD from three independent experiments performed in duplicate. Statistical analysis was performed using unpaired Student's *t*-tests (for EPS) and two-way ANOVA followed by Tukey's multiple comparisons test for biofilm biomass in microcosm biofilm (**p < 0.01, ***p < 0.001 and ****p < 0.0001). (D) FISH and EPS staining of *S. mutans* biofilms at 48 h. Maximum intensity projection confocal images of untreated (top) and 100 µM buprenorphine-treated (bottom) biofilms. All bacteria: EUB338 probe (green); *S. mutans*: MUT590 probe (magenta); EPS: Alexa Fluor 405-dextran (cyan). Merged images illustrate spatial distribution and co-localization. Scale bars = 70 µm.

To evaluate the impact of buprenorphine on complex oral biofilms, we utilized a saliva-derived microcosm model in which HA discs were coated with salivary pellicle and inoculated with fresh human saliva (Figure 5A). The biofilms were grown under static conditions in the presence or absence of 100 μM buprenorphine and analyzed over 24, 48 and 72 h. Crystal violet staining revealed a significant, time-dependent increase in total biomass in the buprenorphine-treated group compared to untreated controls at all time points (Figure 5B). The difference in biomass was most pronounced at 48 and 72 h, indicating a sustained enhancement of biofilm development (****p < 0.0001). Consistent with these findings, quantification of EPS at 48 h showed significantly higher EPS production in buprenorphine-treated biofilms (Figure 5C; ***p < 0.001). These findings suggest that buprenorphine not only enhances early biofilm accumulation but also promotes sustained matrix production within polymicrobial oral communities. Additional spatial and compositional analyses using fluorescence *in situ* hybridization (FISH) revealed that buprenorphine-treated biofilms displayed denser total bacterial biomass (EUB338 probe, green) and more extensive EPS coverage (Alexa Fluor 405-dextran, cyan) compared to untreated controls, with *S. mutans* (MUT590 probe, magenta) more frequently embedded within EPS-rich regions (Figure 5D). Merged channel images highlighted greater overlap among bacterial populations and EPS, suggesting that buprenorphine promotes both biomass expansion and closer integration of *S. mutans* within the extracellular matrix.

### Buprenorphine induces competence and biofilm-associated gene expression in planktonic *S. mutans*

**Figure 6.**
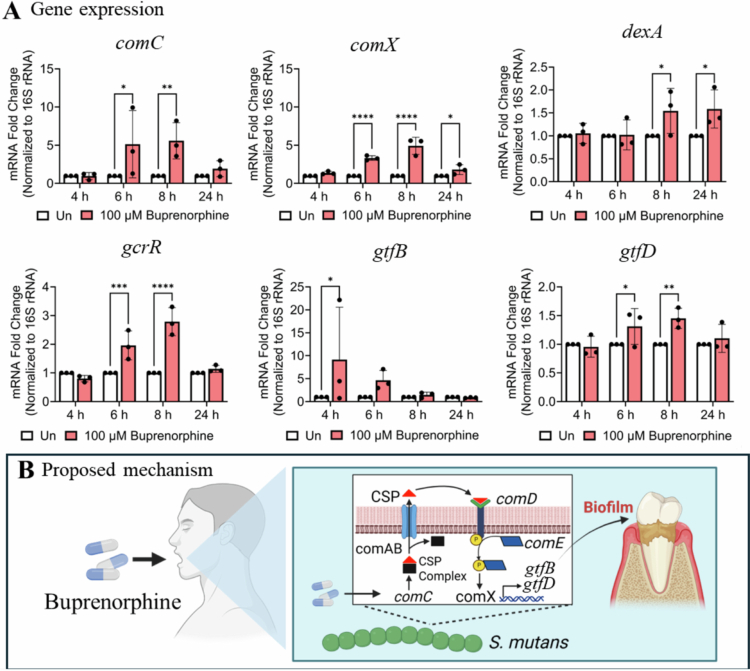
Buprenorphine enhances competence and genes associated with biofilm formation in *planktonic S. mutans.* (A) RT-qPCR analysis of competence (*comC* and *comX*) and biofilm-associated (*dexA*, *gcrR*, *gtfB* and *gtfD*) genes in planktonic cultures of *S. mutans* following treatment with 100 μM buprenorphine. Samples were collected at 4, 6, 8 and 24 h, and expression was quantified relative to that of untreated controls (Un) and normalized to that of 16S rRNA. *comC* encodes the competence-stimulating peptide (CSP) precursor. *comX* is an alternative sigma factor driving late competence gene expression. *gcrR* is a response regulator involved in stress responses and biofilm regulation. *gtfB* and *gtfD* are glucosyltransferase enzymes responsible for synthesizing extracellular polysaccharides (EPS), promoting biofilm formation. The bars represent mean ± SD from three independent biological replicates. Statistical significance was determined by unpaired two-tailed *t*-test: *p* < 0.05; *p* < 0.01; *p* < 0.001 and *p* < 0.0001. (B) Schematic model of the proposed mechanism: buprenorphine exposure enhances CSP signaling via the ComDE pathway, leading to the activation of *com* genes and increased EPS production through *gtfB*/*gtfD*, ultimately promoting *S. mutans* biofilm formation. Created with BioRender.com.

To investigate the effects of buprenorphine on gene regulation in *S. mutans* under planktonic growth conditions, we performed RT-qPCR targeting genes involved in competence signaling and biofilm formation (Figure 6A). Treatment with 100 μM buprenorphine significantly increased the expression of *comC*, which encodes the precursor of the competence-stimulating peptide (CSP), with a marked peak at 6 and 8  h post-treatment (Figure 6A). The expression of *comX*, a key alternative sigma factor that activates late competence genes, was also upregulated at 6 and 8 h. We next assessed genes related to the stress response and biofilm formation. The expression of *gcrR*, a regulatory gene implicated in environmental adaptation and biofilm control, was significantly induced as early as 4 h after buprenorphine exposure. *dexA*, which encodes a dextranase involved in remodeling the biofilm matrix, was also elevated in response to buprenorphine, particularly at later time points. Notably, the expression of the EPS biosynthesis genes *gtfB* and *gtfC*, which encode glucosyltransferases essential for extracellular polysaccharide production and structural integrity of the biofilm matrix, was significantly increased at 6 and 8 h post-treatment. These transcriptional changes occurred in the absence of detectable effects on planktonic growth, indicating that buprenorphine enhances both competence-related and biofilm-associated gene expression in *S. mutans*. A schematic model summarizing the proposed regulatory framework is shown in Figure 6B, linking planktonic transcriptional responses to the enhanced biofilm and EPS phenotypes observed under biofilm conditions.

## Discussion

This study investigated the effects of buprenorphine, a partial μ-opioid receptor agonist commonly used for OUD [1], on the behavior of *S. mutans*, a key cariogenic bacterium in the oral cavity [44,45]. Our findings indicate that while buprenorphine does not impact *S. mutans* growth kinetics, acidogenic potential or acid tolerance, it significantly enhances biofilm formation and EPS production. These effects were accompanied by the upregulation of genes involved in competence signaling (*comC*, *comX*) [46], biofilm regulation (*gcrR*, *dexA*) [47,48] and EPS biosynthesis (*gtfB*, *gtfC*) [37,47,49], indicating that buprenorphine drives a transcriptional program favoring microbial persistence and structural reinforcement of the biofilm matrix. Our data raise important clinical considerations, particularly in light of the increasing use of buprenorphine for long-term OUD treatment. Although buprenorphine does not increase acid production directly, the enhanced capacity of *S. mutans* to form dense, resilient biofilms may still elevate caries risk by promoting more stable colonization and greater resistance to mechanical removal and host defenses. This aligns with emerging epidemiological evidence suggesting a link between opioid use and an increased prevalence of dental caries and oral health complications [6–11,29].

Bacterial growth is a prerequisite for *S. mutans* to form biofilms, as an adequate population density is necessary to establish and maintain biofilm architecture. Since biofilm development relies on active cell division, metabolic activity and extracellular matrix production, any factor that inhibits bacterial growth could confound the interpretation of biofilm-associated phenotypes. Therefore, it was essential to determine whether buprenorphine impacts *S. mutans* viability at physiologically relevant concentrations. Buprenorphine is commonly administered for OUD in sublingual formulations, either alone or combined with naloxone, typically starting at 4–8 mg/day. A recent study [29] reported high accumulation of buprenorphine in the oral cavity of rats following sublingual administration (2 mg/kg), with drug concentrations reaching approximately 1 μM in the salivary glands, 5 μM in the tongue and up to 50–100 μM in the oral fluid, indicating sustained oral exposure rather than a single transient peak. While quantitative human salivary concentration–time data remain limited, clinical studies have demonstrated that a substantial fraction of sublingually administered buprenorphine remains in saliva during the absorption period, supporting biologically plausible oral cavity exposure [50]. Based on these data, we selected a dose range of 0.01–100 μM buprenorphine to model potential exposure levels in the human oral cavity. Under these conditions, we observed no adverse effect on *S. mutans* growth kinetics (Figure 1), indicating that buprenorphine does not exert bacteriostatic or bactericidal effects. This allowed us to investigate the specific effects of this drug on cariogenic traits without confounding influences from impaired bacterial viability.

Acidogenicity is a well-established mechanism by which *S. mutans* contribute to the development of dental caries [16,17]. By fermenting dietary carbohydrates, *S. mutans* produces organic acids that lower the pH of the oral environment, leading to enamel demineralization and cavity formation [13,14,16,17]. Given that buprenorphine did not impact bacterial growth, we next sought to determine whether the drug influences the acidogenic potential of *S. mutans*. To investigate this, we analyzed pH changes in bacterial cultures over time to assess whether buprenorphine affects the acid-production capacity of *S. mutans.* We observed that buprenorphine treatment could not significantly alter the kinetics of acidogenicity by the bacterial strains (Figure 2). The absence of an acidogenicity effect is particularly notable when considered alongside the observed increases in biofilm biomass and EPS production (Figures 4 and 5). Carbohydrate utilization (Figure 3) revealed only minimal differences between the buprenorphine-treated and untreated cultures, with the exception of slightly increased metabolism of D-melibiose and D-cellobiose. While these disaccharides could theoretically contribute to glucan synthesis or acid production under certain nutrient conditions, the changes observed here were modest and may have limited biological relevance in planktonic cultures. The absence of changes in acidogenicity or carbohydrate utilization under buprenorphine treatment is noteworthy, as it suggests that the observed increases in biofilm biomass and EPS production arise from biofilm-specific regulatory shifts rather than alterations in core metabolic activity (Figures 4 and 5). Since acid production is a primary virulence determinant in the planktonic and early biofilm phases [44,51–53], the lack of change here shifts the focus toward structural and ecological mechanisms of enhanced cariogenicity. In other words, buprenorphine does not make *S. mutans* a ‘stronger acid producer’, but it may create a more favorable physical environment for acid retention and localized demineralization by promoting thicker, EPS-rich biofilms. This distinction has clinical importance, as it suggests that interventions targeting matrix accumulation, rather than acid suppression, may be more relevant for mitigating potential buprenorphine-associated increases in caries risk.

Since no differences were observed in bacterial growth dynamics or acidogenicity and the highest concentration of buprenorphine used (100 μM) did not alter the pH of the media, we next examined the effect of buprenorphine on *S. mutans* at different pH levels. This finding aligns with buprenorphine's acidic nature and helps determine whether an acidic environment is required for buprenorphine to exert its effects on bacteria. *S. mutans* is well-adapted to survive and proliferate in acidic environments, a trait known as acid tolerance or aciduricity [16,17]. This capacity is essential for its role in the development of dental caries, allowing the bacterium to maintain metabolic activity and viability despite repeated acid challenges within the dental biofilm [16,17]. Acid tolerance in *S. mutans* is mediated by several mechanisms, including proton extrusion via the F₁F₀-ATPase, alterations in membrane fatty acid composition to reduce proton permeability and the activation of acid stress response regulators that coordinate protective gene expression [54–57]. In the present study, buprenorphine exposure did not impair or enhance *S. mutans* survival, even under conditions of acidic stress (Figure 2). These findings suggest that buprenorphine neither compromises the bacterium's intrinsic acid resistance mechanisms nor exerts additional protective effects, indicating that *S. mutans* aciduricity remains robust despite the presence of this opioid. Additionally, while buprenorphine itself is not chemically acidic, sublingual buprenorphine formulations used clinically are acidic and patients undergoing sublingual buprenorphine therapy often exhibit reduced salivary buffering capacity, thereby increasing their risk for caries [6]. By examining the effects of buprenorphine on *S. mutans* at different pH levels, this study isolates the contribution of environmental acidity from the direct effects of the drug and clarifies whether an acidic environment is necessary for buprenorphine to exert any potential modulatory effects on bacterial physiology.

The most pronounced effects of buprenorphine were observed in the biofilm assays (Figures 4 and 5). In the monospecies model, buprenorphine treatment significantly increased both the total biomass and EPS content without altering the CFU counts (Figure 4). This finding indicates that biofilm thickening was driven primarily by enhanced matrix accumulation rather than increased bacterial proliferation. Biofilms are structured microbial communities attached to a surface and encased in a self-produced matrix [45,49,58]. These genes are a key virulence trait of *S. mutans* because they enable persistence on tooth surfaces and resistance to environmental stress [45,49,58]. Within this matrix, EPS serve as a scaffold that holds bacterial cells together and facilitates adhesion to the tooth surface. EPSs, which are primarily composed of glucans synthesized by glucosyltransferase enzymes, serves multiple critical functions in virulence [59]. First, EPS facilitates the strong adherence of *S. mutans*, allowing for stable colonization and the establishment of structured biofilm communities [58,60,61]. Second, the EPS matrix provides mechanical stability and protection against environmental stressors, such as pH fluctuations and antimicrobial agents, thereby enhancing bacterial survival within the oral cavity [53,62,63]. Additionally, EPS modulates the diffusion of nutrients and metabolic byproducts, creating localized acidic microenvironments that contribute to enamel demineralization and caries development [45,49,53,58,60,62]. Aggregation assays (Figure 1E) showed no difference between treated and untreated cultures, which indicates that the observed biofilm enhancement is not due to increased bacterial clumping in suspension, a process in which cells adhere to one another rather than to a surface but instead reflects matrix-dependent architectural changes.

*In vitro* oral biofilm models, ranging from mono-species to multi-species and microcosm systems, are widely used to investigate biofilm development [64]. While the mono-species model captured the direct effects of buprenorphine on *S. mutans* in isolation, it does not account for the complexity of natural oral communities [64]. Because oral biofilms are highly complex microbial ecosystems containing thousands of bacterial species [44,65], we also performed experiments using a saliva-derived microcosm model, which incorporates diverse microbial interactions and more closely reproduces the microbial diversity and structural heterogeneity observed *in vivo* [34–36,64,66]. This effect was even more evident in this model, where biomass increases were most pronounced at 48 and 72 h and were accompanied by greater EPS production measured at 48 h, as well as closer spatial integration of *S. mutans* within matrix-rich regions (Figure 5). FISH imaging performed at 48 h further confirmed this structural reorganization, revealing that *S. mutans* cells in buprenorphine-treated biofilms were more frequently embedded within EPS-enriched domains, potentially conferring greater mechanical stability and protection from environmental stresses. Taken together, these results suggest that the enhanced biofilm formation and EPS production observed are not driven by broad shifts in central carbohydrate metabolism, but rather by biofilm-associated regulatory changes that foster a microenvironment conducive to biomass and EPS accumulation, potentially increasing the cariogenic potential of *S. mutans* and highlighting the need to consider the effects of opioid medications on microbial diversity as well as host physiology.

To further elucidate the regulatory basis of this phenotype, we examined the transcriptional response of planktonic *S. mutans* to buprenorphine. It is well established that biofilm transcriptional behavior differs from that of planktonic cells of *S. mutans* UA159 [67]. Accordingly, RT-qPCR analyses (Figure 6) were performed in planktonic cultures to capture acute, direct regulatory responses to buprenorphine under homogeneous exposure conditions. The biological relevance of these transcriptional changes was subsequently evaluated using complementary biofilm and EPS assays, which represent the primary phenotypic outcomes of this study (Figures 4 and 5). Our results revealed that buprenorphine significantly alters the transcriptional landscape of planktonic *S. mutans*, shifting the expression of genes associated with competence signaling and biofilm development. While these transcriptional changes were measured in planktonic cultures, future studies directly profiling transcriptomes within biofilm-grown *S. mutans* will be important to define how buprenorphine modulates gene expression.

In *S. mutans*, competence refers to a transient physiological state in which bacteria become capable of taking up and integrating exogenous DNA from their surroundings [46,68,69]. Beyond facilitating genetic exchange, the competence signaling network also coordinates stress responses and regulates traits important for biofilm development, including extracellular matrix production and cell–cell communication [69] and this process is largely controlled by quorum sensing [46,68,69]. In brief, in *S. mutans*, the *comC* gene encodes the precursor of the competence-stimulating peptide (CSP), which is secreted and accumulates in the extracellular environment as the population grows. Once the CSP reaches a threshold concentration, it binds to the ComD histidine kinase receptor, triggering phosphorylation of the response regulator ComE and subsequent activation of *comX*. The *comX* gene encodes an alternative sigma factor that drives the expression of late competence genes, many of which are involved in DNA uptake, stress tolerance and biofilm maturation [46,68,69]. The observed upregulation of *comC* and *comX* in buprenorphine-treated cultures therefore suggests that the drug enhances quorum-sensing activity, potentially leading to coordinated increases in EPS production and structural reinforcement of the biofilm matrix (Figure 6).

The concurrent increase in both *comC* and *comX* at 6 and 8 h post-treatment indicates the activation of the full competence regulatory cascade, which may facilitate horizontal gene transfer and adaptive responses under buprenorphine exposure. In addition to competence, buprenorphine also modulated genes involved in biofilm formation and stress adaptation. Notably, *gcrR*, a transcriptional regulator linked to acid tolerance, the oxidative stress response and the repression of glucosyltransferase genes [48], was upregulated as early as 4 h. The early induction of *gcrR* may reflect a generalized stress response or a regulatory shift toward a more sessile, biofilm-associated phenotype. The expression of *dexA*, which encodes a dextranase that degrades α-1,6-linked glucans [47], was also elevated. While *dexA* is classically associated with matrix remodeling, its increased expression in this context may reflect dynamic regulation of the biofilm structure, possibly to balance matrix accumulation with dispersal. Importantly, buprenorphine significantly induced the expression of *gtfB* and *gtfC*, two critical glucosyltransferase genes responsible for the synthesis of water-insoluble and soluble glucans, respectively [37,47,49]. These exopolysaccharides are essential for the development and stability of the *S. mutans* biofilm matrix. The upregulation of *gtfB* and *gtfC* suggests that buprenorphine promotes an extracellular environment conducive to strong surface adhesion and intercellular aggregation, both of which are hallmarks of cariogenic biofilms.

Patients under drug abuse face a heightened risk for oral health complications due to xerostomia, high dietary sugar intake, irregular oral hygiene practices and limited access to preventive dental care [23,24,70]. Our findings add a new dimension to this risk profile by demonstrating that buprenorphine can alter the transcriptional program of *S. mutans* in ways that favor robust biofilm development. Specifically, the drug-upregulated genes linked to quorum sensing (*comC*, *comX*), stress adaptation (*gcrR*) and extracellular polysaccharide synthesis (*gtfB*, *gtfC*) collectively driving the formation of thicker, EPS-rich biofilms. In a clinical context, such biofilms are more resistant to salivary clearance, mechanical disruption and antimicrobial agents, allowing them to persist on tooth surfaces and maintain a cariogenic microenvironment. These drug-induced microbial changes could interact synergistically with other OUD-related risk factors to accelerate caries progression. For example, reduced salivary flow and frequent intake of fermentable carbohydrates can enhance acid production within biofilms, while increased EPS production may trap acids near the tooth surface, amplifying demineralization risk. Recognizing that medications like buprenorphine may influence the behavior of cariogenic bacteria underscores the importance of a multidisciplinary approach in OUD management. Dental professionals, addiction specialists and primary care providers should coordinate to ensure regular oral health assessments, targeted preventive measures and patient education on dietary habits and oral hygiene for individuals receiving long-term buprenorphine therapy.

While this work provides important mechanistic insights, some limitations should be considered when interpreting the findings. First, the experiments were conducted mainly using *in vitro* mono-species and saliva-derived biofilm models, which do not fully replicate the complexity of the oral environment *in vivo*. While mono-species biofilms offer valuable mechanistic insights, they lack the microbial diversity and interspecies interactions characteristic of natural dental biofilms. In contrast, saliva-derived biofilms are more physiologically relevant, as they incorporate a heterogeneous microbial community originating from the host's oral microbiota, better reflecting the structural and functional complexity of biofilms that form in the oral cavity [34–36]. However, even with this increased physiological relevance, important host factors such as immune responses, salivary flow and composition, dietary influences and oral hygiene practices were not replicated in the *in vitro* setting [34–36], all of which can significantly influence *S. mutans* behavior and biofilm dynamics in clinical contexts. Second, while we used buprenorphine at physiologically relevant concentrations, the actual levels of the drug present in the oral cavity during treatment, particularly with different administration routes, such as sublingual tablets or films, remain poorly defined. Lastly, this study focused specifically on *S. mutans*, and the impact of buprenorphine on other oral microorganisms and overall microbiome structure remains unknown. The underlying molecular mechanisms through which buprenorphine enhances biofilm formation and EPS production also warrant further investigation.

Future studies incorporating multi-species biofilm models, clinical isolates and *in vivo* systems are essential to validate these findings. The inclusion of *S. mutans* clinical isolates obtained from individuals receiving buprenorphine therapy would allow direct assessment of strain-specific adaptations and phenotypic variability in a clinically relevant context. Multi-species biofilm models would allow investigation of how buprenorphine influences microbial community composition, interspecies interactions and overall biofilm pathogenicity. In parallel, *in vivo* approaches, including animal models and clinical cohorts, will be critical to assess how host factors such as immune responses, salivary flow and oral hygiene practices modulate these effects under physiological conditions. At the molecular level, broader transcriptomic and metabolic analyses will be necessary to define the global regulatory pathways underlying buprenorphine-enhanced biofilm formation and EPS production. Finally, future investigations should evaluate whether targeted preventive strategies, such as enhanced oral hygiene protocols, salivary substitutes or antimicrobial agents, can mitigate the biofilm-promoting effects of buprenorphine and reduce caries risk in individuals undergoing long-term opioid therapy. Collectively, these future investigations will strengthen the translational relevance of the present findings and inform evidence-based strategies for managing caries risk in patients undergoing long-term buprenorphine treatment.

## Conclusions

Buprenorphine, a widely prescribed therapy for opioid use disorder, does not alter *S. mutans* growth, viability, acidogenicity, acid tolerance, or carbohydrate metabolism in planktonic cultures. Instead, it significantly increased biofilm biomass and extracellular polysaccharide production in both the monospecies and saliva-derived microcosm models. These structural changes coincide with the upregulation of genes linked to quorum sensing (*comC, comX*), stress adaptation (*gcrR*) and matrix synthesis (*gtfB, gtfC*), indicating that buprenorphine activates a biofilm-specific regulatory program that favors matrix accumulation and spatial integration. By fostering denser, EPS-rich biofilms, buprenorphine may contribute to a more cariogenic oral environment, compounding the elevated risk of dental disease already observed in patients undergoing opioid therapy. These findings emphasize the need to consider microbial responses when evaluating the long-term effects of therapeutic opioids and highlight the importance of preventive oral health strategies for individuals receiving buprenorphine treatment.

## Data Availability

All relevant data are provided within this paper, further details are available upon request.
